# Antenatal iron supplementation, FGF23, and bone metabolism in Kenyan women and their offspring: secondary analysis of a randomized controlled trial

**DOI:** 10.1093/ajcn/nqaa417

**Published:** 2021-03-01

**Authors:** Vickie S Braithwaite, Martin N Mwangi, Kerry S Jones, Ayşe Y Demir, Ann Prentice, Andrew M Prentice, Pauline E A Andang'o, Hans Verhoef

**Affiliations:** Medical Research Council (MRC) Nutrition and Bone Health Research Group, Clifford Allbutt Building, University of Cambridge, Cambridge Biomedical Campus, Cambridge, CB2 0AH, United Kingdom (formerly the MRC Elsie Widdowson Laboratory, 120 Fulbourn Road, Cambridge, CB1 9NL, United Kingdom); Medical Research Council (MRC) Epidemiology Unit, University of Cambridge School of Clinical Medicine, Cambridge Biomedical Campus, Cambridge, CB2 0QQ, United Kingdom; Wageningen University, Division of Human Nutrition and Health, P.O. Box 17, 6700 AA Wageningen, The Netherlands; University of Malawi, College of Medicine, Training and Research Unit of Excellence, Private Bag 360, BT 3, Chichiri, Blantyre, Malawi; National Institute for Health Research (NIHR) Biomedical Research Centre Nutritional Biomarker Laboratory, MRC Epidemiology Unit, University of Cambridge, Cambridge Biomedical Campus, Cambridge, CB2 0AH, United Kingdom; Meander Medical Centre, Laboratory for Clinical Chemistry and Hematology, P.O. Box 1502, 3800 BM Amersfoort, The Netherlands; Medical Research Council (MRC) Nutrition and Bone Health Research Group, Clifford Allbutt Building, University of Cambridge, Cambridge Biomedical Campus, Cambridge, CB2 0AH, United Kingdom (formerly the MRC Elsie Widdowson Laboratory, 120 Fulbourn Road, Cambridge, CB1 9NL, United Kingdom); Medical Research Council (MRC) Unit The Gambia at London School of Hygiene & Tropical Medicine, Atlantic Boulevard, Fajara, Banjul, The Gambia; Medical Research Council (MRC) Unit The Gambia at London School of Hygiene & Tropical Medicine, Atlantic Boulevard, Fajara, Banjul, The Gambia; Maseno University, School of Public Health and Community Development, Maseno, Kenya; Wageningen University, Division of Human Nutrition and Health, P.O. Box 17, 6700 AA Wageningen, The Netherlands; Wageningen University, Cell Biology and Immunology Group, P.O. Box 338, 6708 WD Wageningen, The Netherlands

**Keywords:** iron deficiency anemia, fibroblast growth factor (FGF23), phosphate, vitamin D, bone, Africa, pregnancy

## Abstract

**Background:**

Fibroblast growth factor-23 (FGF23) regulates body phosphate homeostasis primarily by increasing phosphaturia. It also acts as a vitamin D-regulating hormone. Maternal iron deficiency is associated with perturbed expression and/or regulation of FGF23 and hence might be implicated in the pathogenesis of hypophosphatemia-driven rickets in their offspring.

**Objectives:**

We aimed to determine the effect of antenatal oral iron supplementation on FGF23 concentration and maternal and infant markers of bone-mineral regulation.

**Methods:**

We performed a secondary analysis of a trial in which 470 rural Kenyan women with singleton pregnancies and hemoglobin concentrations ≥ 90 g/L were randomly allocated to daily, supervised supplementation with 60 mg elemental iron as ferrous fumarate or placebo from 13–23 weeks of gestation until 1 mo postpartum. As previously reported, iron supplementation improved iron status in mothers and neonates. For the present study, we reanalyzed all available plasma samples collected in mothers and neonates at birth, with primary outcomes being concentrations of FGF23, measured by 2 assays: 1 that detects intact hormone and C-terminal cleavage products (total-FGF23) and another that detects the intact hormone only (intact-FGF23).

**Results:**

Analysis was performed on 433 women (*n* = 216, iron group; *n* = 217, placebo group) and 414 neonates (*n* = 207, iron group; *n* = 207, placebo group). Antenatal iron supplementation reduced geometric mean total-FGF23 concentrations in mothers and neonates by 62.6% (95% CI: 53.0%, 70.3%) and 15.2% (95% CI: −0.3%, 28.4%, *P* = 0.06), respectively. In addition, it increased geometric mean neonatal intact-FGF23 concentrations by 21.6% (95% CI: 1.2%, 46.1%), increased geometric mean maternal hepcidin concentrations by 136.4% (95% CI: 86.1%, 200.3%), and decreased mean maternal 25-hydroxyvitamin D concentrations by 6.1 nmol/L (95% CI: −11.0, −1.2 nmol/L).

**Conclusions:**

Analysis of this randomized trial confirms that iron supplementation can reverse elevated FGF23 production caused by iron deficiency in iron-deficient mothers and their neonates. Further investigations are warranted to assess to what extent iron supplementation can prevent FGF23-mediated hypophosphatemic rickets or osteomalacia.

## Introduction

**FIGURE 1:**
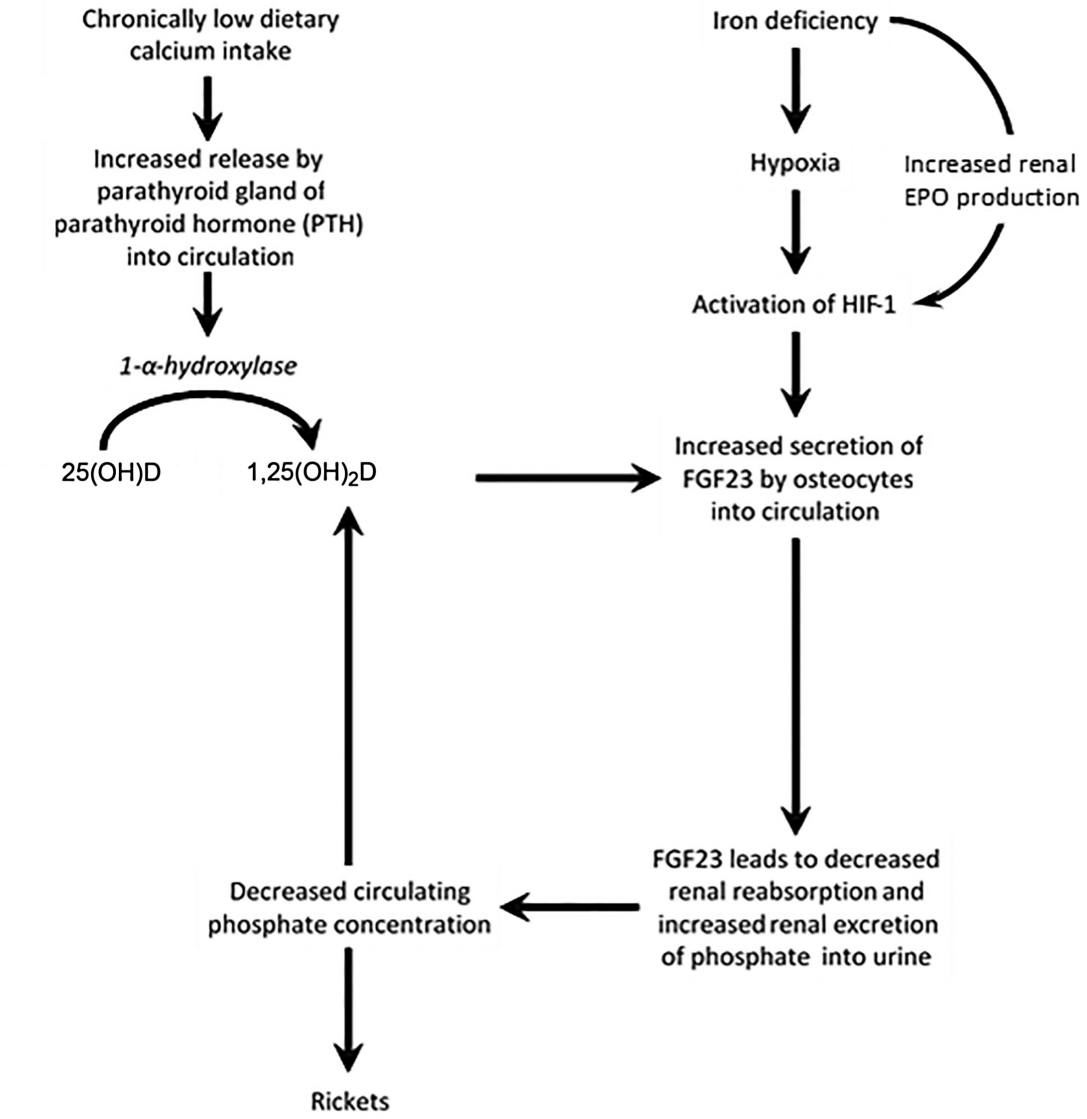
Putative mechanisms whereby deficiencies of iron and calcium lead to hypophosphatemia and rickets. FGF23 is a hormone that is mainly secreted by osteocytes in response to elevated concentrations of 1,25(OH)_2_D. FGF23 causes the internalization of sodium phosphate cotransporters in the proximal tubule of the kidney, inhibiting 1-α-hydroxylase [which catalyzes the hydroxylation of 25(OH)D to 1,25(OH)_2_D] and stimulating expression of 24-hydroxylase [which initiates the degradation of 1,25(OH)_2_D to an inactive form of vitamin D]. The net effect is an increased phosphate loss in the urine, an accompanying reduction in circulating phosphate concentration, and a decrease in 1,25(OH)_2_D. Iron deficiency probably stimulates FGF23 gene expression by activating HIF-1 (7, 13), the master transcriptional regulator of cellular and developmental responses to hypoxia. Iron deficiency also increases renal EPO production. EPO mediates the relation between HIF-1 and FGF23 resulting in further increases in FGF23 gene expression (14). In osteocytes, a proportion of FGF23 is normally broken down by proteolytic cleavage before secretion, resulting in both intact, biologically active hormone and C-terminal and N-terminal fragments (with unclear biological activity) in the circulation (15). Some studies in healthy adults have indicated that low serum iron was associated with elevated total-FGF23 but not intact-FGF23, suggesting that cleavage maintains phosphate homeostasis despite increased FGF23 expression (15, 16), whereas others have shown that low serum iron is associated with elevated total- and intact-FGF23 (17, 18). EPO, erythropoietin; FGF23, fibroblast growth factor-23; HIF-1, hypoxia inducible factor 1; intact-FGF23, intact fibroblast growth factor-23; total-FGF23, intact and C-terminal fragments of fibroblast growth factor-23; 1,25(OH)_2_D, 1,25-dihydroxyvitamin D; 25(OH)D, 25-hydroxyvitamin D.

There is increasing evidence that iron deficiency in children with chronically inadequate calcium intake may predispose to rickets, one of the most common noncommunicable childhood diseases in developing countries (1). In this pathogenic pathway (**Figure 1**), there seems to be a central role for fibroblast growth factor-23 (FGF23), a key regulator of circulating concentrations of phosphate and 1,25-dihydroxyvitamin D [1,25(OH)_2_D—the bioactive form of vitamin D] (2). In vitamin D-replete Gambian children, rickets was associated with elevated plasma concentrations of total-FGF23 (as measured by an assay which detects both the intact hormone and the C-terminal fragments that result from its cleavage), 1,25(OH)_2_D, and total alkaline phosphatase (a marker of dysregulated bone formation that is elevated in rickets and osteomalacia) and with lower plasma concentrations of phosphate (3). Gambian children with a history of rickets-like bone deformities had a higher prevalence of anemia than community controls, and total-FGF23 concentration was negatively associated with hemoglobin concentration (4). In an iron supplementation study among Gambian children, plasma total-FGF23 concentration at baseline was inversely associated with markers of iron (plasma ferritin concentration, hemoglobin concentration), whereas at 3 mo after the start of iron supplementation, plasma total-FGF23 concentrations were lower than at the start of the intervention (5). As with most such studies, ethics constraints precluded the use of a placebo control group. In a prospective cohort study, Gambian children born to mothers who were iron deficient during pregnancy had higher concentrations of total-FGF23 and total alkaline phosphatase than those whose mothers had normal iron status and this difference persisted ≤2 y of age (6). In mice, diet-induced iron deficiency during pregnancy and nursing resulted in elevated serum concentrations of total-FGF23 and intact-FGF23 (as measured using an assay which only detects the intact hormone), hypophosphatemia, and reduced serum concentrations of 1,25(OH)_2_D in their pups (7). These data suggest that maternal iron deficiency may lead to perturbations in FGF23 regulation that may in turn lead to hypophosphatemia-driven rickets in the offspring. In addition, maternal bone mineral density decreases by 3%–5% during pregnancy and lactation (8). This physiological change, coupled with iron-deficiency phosphate loss, may put additional pressures on the maternal skeleton, thus predisposing iron-deficient women to poor bone health in later life.

*FGF23* gene expression occurs predominantly within osteocytes but the FGF23 hormone regulates circulating phosphate concentrations via the FGF receptor in renal tubule cells where FGF23 signaling causes a reduction in phosphate resorption from the glomerular filtrate and thus renal phosphate excretion. The intact FGF23 hormone undergoes proteolytic cleavage into its C- and N-terminal fragments. Although the intact FGF23 hormone is known to have effects on phosphate and vitamin D metabolism, there is conflicting evidence as to whether the C-terminal FGF23 fragment is active, acts in the same way (9), or acts in the opposite way to the intact hormone (10).

The requirement for iron and the demands on the kidney increase during pregnancy, and the risk of developing iron deficiency and/or anemia increases substantially. Iron absorption and thus the effect of iron interventions on systemic biomarkers (e.g., hemoglobin concentration) are known to depend on iron status. This process is mediated by circulating concentrations of hepcidin, the master regulator of body iron homeostasis. Causality of the relations between iron deficiency and bone metabolism has not yet been shown, because evidence has so far been obtained exclusively from observational studies and animal studies. Such an effect would be pertinent to developing countries, where iron deficiency often affects the majority of pregnant women and a substantial proportion of young children (11).

The aim of this current study was to investigate the effect of antenatal oral iron supplementation on circulating concentrations of FGF23 and hence on markers of phosphate and bone metabolism (including vitamin D) and kidney function at birth in pregnant Kenyan women and their offspring. In addition, we explored to what extent the response of these markers to intervention depended on initial iron status.

## Methods

### Study design, participants, randomization, and blinding

The present study used samples and data that were collected as part of a randomized placebo-controlled trial in rural Kenya (NCT01308112) originally designed to measure the effect of antenatal iron supplementation on maternal *Plasmodium* infection risk, maternal iron status, and neonatal outcomes. The recruitment for the original trial was conducted between 2011 and 2013. Study details and main results have been published elsewhere (12). In brief, pregnant women aged 15–45 y with singleton pregnancies, gestational age of 13–23 wk (determined by obstetric ultrasonography), and hemoglobin concentration ≥ 90 g/L were individually randomly assigned at a 1:1 allocation ratio to 60 mg of elemental iron as ferrous fumarate or placebo until 1 mo postpartum. The supplements contained no other micronutrients and their neonates did not receive any additional supplements. From screening until the end of the intervention, local mill operators added fortificant iron (target dose: 20 mg/kg flour) to grain routinely brought for milling by homestead members of all the participating women. Based on weighed intake studies, we estimate that fortification supplied on average 5.7 mg of elemental iron as ferric sodium ethylenediaminetetraacetate daily to the pregnant women in both arms of the intervention. Participants and field staff were blinded to the randomization and intervention until data analysis.

All women provided written informed consent. Approval for the original study was obtained from ethics committees at the London School of Hygiene and Tropical Medicine, United Kingdom, and the Kenyatta National Hospital/University of Nairobi (ethics approval number KNH-ERC/A/453, 6/01/2010); additional approval was obtained from the latter committee (KNH-ERC/A/272, P280/05/2017) to perform additional analyses on archived plasma samples to generate the data presented in this report.

### Procedures

Maternal weight and height were measured at baseline, and neonatal weight, length, and head circumference were measured at birth. At baseline, we collected venous blood samples in EDTA-coated tubes from women. We also collected maternal venous blood, neonatal cord blood, and placental biopsies within 1 h postpartum. For home deliveries, samples were collected within 2 h postpartum.

Plasma was stored within 2 h of blood collection in liquid nitrogen (−196°C) and subsequently frozen (−80°C) until analysis. We performed ELISA tests at the Medical Research Council Elsie Widdowson Laboratory, Cambridge, United Kingdom to measure the following markers in plasma collected at delivery: total-FGF23 using the C-terminal assay (60-6100) which detects both the C-terminal fragment and the intact hormone, and intact-FGF23 using the intact-FGF23 assay (60-6600) which detects only the intact form of the hormone (Immutopics); 1,25(OH)_2_D (the active metabolite of vitamin D; Immunodiagnostic Systems); β-C-terminal telopeptide (β-Crosslaps—a marker of bone resorption that is elevated in increased bone resorption; Immunodiagnostic Systems); and hepcidin (a regulator of iron metabolism that is low in iron deficiency and raised by inflammation; Hepcidin-25, Bioactive). Hemoglobin concentration was measured in the field by photometer (HemoCue 301, Radiometer) and plasma α_1_-acid glycoprotein (a marker of inflammation) and soluble transferrin receptor (sTfR—a marker of iron status) were measured at Meander Medical Centre, Amersfoort, Netherlands (UniCel DxC 880i analyzer, Beckman Coulter) as part of the original trial (12). Plasma concentrations of ferritin, C-reactive protein (CRP—a marker of inflammation), 25-hydroxyvitamin D [25(OH)D—marker of vitamin D status], total alkaline phosphatase, intact parathyroid hormone (PTH—a primary regulatory hormone of calcium that is elevated in calcium deficiency), cystatin C (a marker that is elevated in kidney dysfunction), and phosphate were measured at Meander Medical Centre, Amersfoort, Netherlands (Architect c16000 and Architect i2000SR, Abbott). ELISA assay accuracy was monitored across the working range of assays using kit controls supplied by the manufacturers. The UK laboratory was accredited by the Vitamin D External Quality Assessment Scheme (http://www.deqas.org/) and the UK National External Quality Assessment Service (https://ukneqas.org.uk/) and the laboratory in the Netherlands by the External Quality Assurance System (https://www.eurl-ar.eu/eqas.aspx) and German Society for Clinical Chemistry and Laboratory Medicine (DGKL) (https://www.dgkl.de/en/). In addition, an aliquot of a pooled plasma sample was assayed in each batch to monitor possible drift in measurements over time. Intra- and interassay CVs were <7% and <6% for total-FGF23, intact-FGF23, β-Crosslaps, 1,25(OH)_2_D, 25(OH)D, PTH, ferritin, CRP, phosphate, total alkaline phosphatase, and cystatin C and <5% and <17% for hepcidin, respectively.

Estimated glomerular filtration rate (eGFR—a measure of kidney glomerular function) was calculated using equations approved by the National Kidney Foundation (https://www.kidney.org/professionals/KDOQI/gfr)—the Chronic Kidney Disease Epidemiology Collaboration (CKD-EPI) cystatin C equations for those aged >18 y: eGFR, mL·min^−1^·1.73 m^−2^ = 123.9 × [cystatin C (mg/L)/0.8]^−1.328^ × 0.996^age^ and for those aged ≤18 y: eGFR, mL·min^−1^·1.73 m^−2^ = 70.69 × [cystatin C (mg/L)]^−0.931^. At baseline, dipstick tests (Access Bio) were used to detect histidine-rich protein-2 and lactate dehydrogenase specific either to *P. falciparum* or to nonfalciparum human *Plasmodium* species (12). qPCR was used to detect *P. falciparum–*specific DNA in erythrocytes, and mothers were also tested for HIV infection. HIV-infected mothers continued or were offered antiretroviral treatment as part of their standard clinical care.

### Outcomes

The primary analysis concerned group differences in plasma concentrations of total-FGF23 and intact-FGF23 in maternal blood samples at delivery and neonatal cord blood samples. As secondary outcomes, we studied other biomarkers of bone metabolism and kidney function, namely, plasma concentrations of phosphate, PTH, total alkaline phosphatase, β-Crosslaps, 25(OH)D, 1,25(OH)_2_D, cystatin C, and eGFR.

### Statistical analysis

Sample size requirements were calculated based on the effect of iron supplementation on *Plasmodium* infection risk (12) as part of the original trial design; because they are not relevant to the current study, they are not reported here. The current study performed analysis on all available plasma samples from mothers at birth (*n* = 433) and from infant cord blood (*n* = 414) at delivery.

Anthropometric *z* scores for the neonates at birth were derived with Kenyan children as a reference (19). The following definitions were used: prematurity–being born at or before 37 completed weeks of gestation (<259 days of gestation as calculated by early pregnancy ultrasound); anemia: hemoglobin concentration < 110 g/L for pregnant women (19); iron deficiency (depleted iron stores)–plasma ferritin concentration <15 µg/L for women and <12 µg/L for neonates (20); inflammation–plasma CRP > 10 mg/L (21); vitamin D insufficiency–25(OH)D concentration <50 nmol/L; and vitamin D deficiency–25(OH)D concentration <30 nmol/L (22). There are no established thresholds for intact-FGF23 or total-FGF23 concentrations or for eGFR in pregnancy and neonates, or for anemia in neonates, and so these variables were not dichotomized. *Plasmodium* infection was defined as past or present maternal infection assessed at parturition, regardless of species, as indicated by ≥1 positive test results for the presence of *Plasmodium* lactate dehydrogenase or *P. falciparum–*specific histidine-rich protein-2 in plasma or by placental histopathology or *P. falciparum* DNA in maternal erythrocytes from venous or placental blood by a PCR test.

Statistical analysis was performed using Stata version 16.0 (StataCorp). We visually inspected histograms to assess the shape of the distribution and to identify possible outliers. Skewed data were normalized by log transformation as appropriate. Groups were described using mean ± SD for normally distributed data and geometric mean ± geometric SD (GSD) for log-transformed data. GSD was calculated as the exponentiated SD of the log-transformed variable. It is a dimensionless factor that indicates variation that is equivalent to subtraction or addition of 1 SD on a log-transformed scale. For plasma CRP concentration at baseline, we computed descriptive statistics with a Tobit model to account for data being left-censored at the limit of quantification (1 mg/L).

Plasma concentrations of ferritin and soluble transferrin receptor (sTfR) are affected by infection and inflammation independently of iron status. We used multiple regression models to adjust the iron markers for such effects (**Supplemental Methods**). We used adjusted plasma iron markers to calculate the body iron index, i.e., the ln of the ratio of the adjusted ferritin concentration to the adjusted sTfR concentration. This indicator has been shown to be linearly associated with quantitative estimates of the size of the body iron store in iron-replete adults, and with the size of the functional deficit that would need to be corrected before iron could again be accumulated in the store in iron-deficient individuals (23).

Crude intervention effects on continuous outcomes were estimated by simple linear regression. To assess the potential role of confounding due to imbalances in baseline variables, we used multiple fractional polynomial regression to estimate intervention effects adjusted for maternal characteristics assessed at randomization, i.e., hemoglobin concentration, body iron index, age, BMI, gestational age at delivery (calculated from early pregnancy ultrasonography), parity, HIV infection, and *Plasmodium* infection. Both in simple linear regression models and in fractional polynomial models, we accounted for heteroscedasticity of the error terms as appropriate (*P* values for Breusch–Pagan/Cook–Weisberg tests < 0.05). Intervention effects are reported as absolute differences in means for normally distributed outcomes, or as relative differences in geometric means for log-transformed outcomes.

For unadjusted prevalence differences, we used Newcombe's method to estimate 95% CIs and “N−1” chi-square tests to compute *P* values (24). We used log-binomial regression models (*adjrr* command in Stata package *st0306.pkg*) to estimate prevalence differences adjusted for baseline characteristics, and to estimate unadjusted prevalence differences when contingency tables contained cells with expected values <10.

In a preplanned analysis, we used multiple fractional polynomial regression analysis to explore to what extent iron status at baseline modified the magnitude of the effect of iron supplementation on FGF23 and selected markers of bone metabolism at delivery, anticipating that iron absorption and thus the response to administered iron would be larger in iron-deficient women than in their iron-replete peers. We examined such effect modification with a single independent variable (body iron index) and 10 outcomes (see the Outcomes section), in both mothers and neonates, which resulted in 20 analyses. We used the *mfpi* procedure in Stata with the “*flex*(3)” specification to define the flexibility of the main effects and interaction models with adjustment for potentially influential maternal characteristics assessed at randomization, i.e., hemoglobin concentration, BMI, gestational age, parity, HIV infection, and *Plasmodium* infection. We used a nominal significance level of 0.05 for selection of variables and power functions; selection of linear, first-degree, or second-degree polynomials was based on the lowest value for Akaike's information criterion. To check for possible overfitting in the interaction models, we examined to what extent possible trends in intervention effects observed across quintiles of body iron index were consistent with effect modification as measured by fractional polynomial regression.

## Results

**FIGURE 2:**
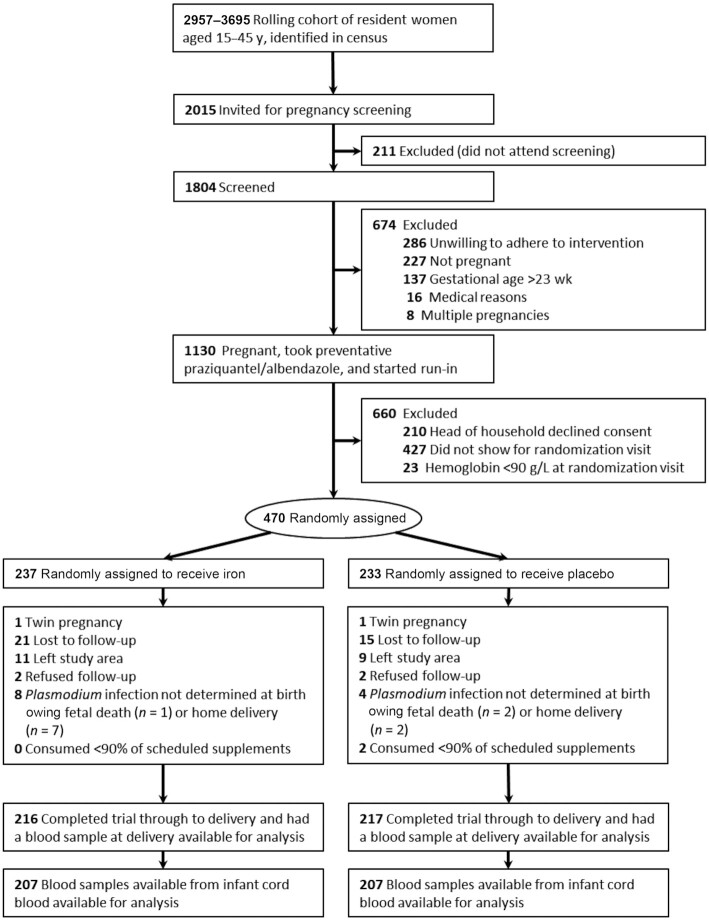
Flow-through of participants in the study and blood samples available for analysis.

Primary outcome data were available for 433 women (92% of 470 women randomly assigned to the intervention). Sample sizes <433 reported in the subsequent sections were due to insufficient plasma volume being available for biochemical analysis (**Figure 2**).

### Maternal and neonatal biomarkers

At baseline, women had a mean age of 25.1 y, 16.2% were nulliparous, 38.3% were anemic, 99.1% were iron deficient (plasma ferritin concentration < 15 µg/L; values adjusted for inflammation and *Plasmodium* infection), 34.4% had a current or recent *Plasmodium* infection, and 21.7% were HIV-infected. At baseline, anthropometric indexes, iron status, and infection status were similar between intervention groups (**Table 1**).

**TABLE 1:** Maternal characteristics at baseline, by intervention group^1^

Characteristic	Placebo (*n* = 217)	Iron (*n* = 216)
Age, y	25.0 ± 6.1	25.1 ± 6.1
Parity,^2^*n*	2 (0–10)	2 (0–9)
Nullipara	19.8 [43]	12.5 [27]
Height, cm	162.4 ± 6.8	162.5 ± 5.9
Weight, kg	57.4 ± 7.4	58.0 ± 7.5
Gestational age at randomization,^3^ wk	17.5 ± 1.2	17.9 ± 1.2
Hemoglobin concentration, g/L	112.4 ± 11.8	113.4 ± 10.7
Anemia (hemoglobin concentration < 110 g/L)	40.5 [88]	36.1 [78]
Plasma ferritin concentration,^3^ µg/L	15.9 ± 2.5	16.3 ± 2.4
Iron deficiency (plasma ferritin concentration < 15 µg/L)		
Without adjustment for inflammation and infection^4^	53.5 [116]	53.7 [116]
With adjustment for inflammation and infection^4^	99.5 [216]	98.6 [213]
Plasma CRP concentration,^3^, ^5^ mg/L	4.1 ± 3.72	4.0 ± 3.79
Plasma AGP concentration, g/L	0.78 ± 0.27	0.76 ± 0.26
Inflammation (plasma CRP ≥ 10 mg/L)	26.7 [58]	23.6 [51]
Current *Plasmodium* infection	36.4 [79]	32.4 [70]
HIV infection	21.7 [47]	21.8 [47]

1 Values are mean ± SD or percentage [*n*] unless otherwise indicated. AGP, α_1_-acid glycoprotein; CRP, C-reactive protein.

2 Median (range).

3 Geometric mean ± geometric SD.

4 Adjustment based on plasma CRP concentration, plasma AGP concentration, and *Plasmodium* infection (see Supplemental Methods).

5 Based on log-transformed data and a Tobit model to account for left-censoring at the limit of quantification (1 mg/L).

Antenatal oral iron supplementation reduced the geometric mean maternal concentration of total-FGF23 (by −62.6%) and mean maternal concentration of 25(OH)D (by −6.1 nmol/L) (**Table 2**). It also improved maternal iron status as shown by an increased geometric mean concentration of hepcidin (by 136.4%); increased mean concentration of hemoglobin (by 9.0 g/L); increased geometric mean concentration of ferritin (by 95.6%); and a reduced prevalence of anemia (by 29.1%) and iron deficiency (by 27.1%).

**TABLE 2:** Effect of antenatal iron supplementation on maternal and neonatal biomarkers at birth^1^

	Maternal blood				Neonatal (cord) blood			
Biomarker concentration or status	*n*	Mean ± SD	Effect (95% CI)	*P* value	*n*	Mean ± SD	Effect (95% CI)	*P* value
Total-FGF23,^2^ RU/mL								
Placebo	217	370.3 ± 3.62	Reference		207	647.3 ± 2.29	Reference	
Iron	216	138.4 ± 3.09	−62.6% (−70.3%, −53.0%)	<0.0005	207	548.6 ± 2.50	−15.2% (−28.4%, 0.3%)	0.06
Intact-FGF23,^2^ ng/L								
Placebo	217	41.3 ± 1.54	Reference		205	6.1 ± 2.65	Reference	
Iron	216	39.4 ± 1.46	−4.5% (−11.5%, 3.1%)	0.24	200	7.4 ± 2.47	21.6% (1.2%, 46.1%)	0.04
25-hydroxyvitamin D, nmol/L								
Placebo	217	99.6 ± 28.7	Reference		204	63.1 ± 19.3	Reference	
Iron	216	93.5 ± 23.0	−6.1 (−11.0, −1.2)	0.01	206	61.5 ± 17.7	−1.6 (−5.2, 2.0)	0.37
Inadequate 25-hydroxyvitamin D (<50 nmol/L)								
Placebo	217	2.8 [6]	Reference		204	23.5 [48]	Reference	
Iron	216	3.7 [8]	0.9% (−3.0%, 5.0%)	0.96	206	25.2 [52]	−1.7% (−10.0%, 6.6%)	0.97
1,25-dihydroxyvitamin D, pmol/L								
Placebo	203	351.0 ± 78.2	Reference		195	203.0 ± 62.9	Reference	
Iron	200	351.1 ± 84.4	0.1 (−15.8, 16.0)	0.98	195	203.9 ± 52.7	0.8 (−10.6, 12.4)	0.88
Parathyroid hormone,^2^ pmol/L								
Placebo	217	4.0 ± 1.94	Reference		205	0.5 ± 2.69	Reference	
Iron	216	3.9 ± 1.92	−2.0% (−13.4%, 10.9%)	0.75	206	0.5 ± 2.97	−3.3% (−21.0%, 18.3%)	0.74
Phosphate, mmol/L								
Placebo	217	1.27 ± 0.32	Reference		205	2.05 ± 0.64	Reference	
Iron	216	1.29 ± 0.24	0.03 (−0.03, 0.08)	0.30	206	2.11 ± 0.65	0.06 (−0.07, 0.18)	0.38
Total alkaline phosphatase,^2^ U/L								
Placebo	217	102.3 ± 1.88	Reference		205	17.3 ± 1.74	Reference	
Iron	215	96.1 ± 1.86	−6.0% (−16.5%, 5.7%)	0.30	206	17.6 ± 2.22	2.6% (−10.2%, 10.2%)	0.77
β-Crosslaps,^2^ µg/L								
Placebo	210	0.6 ± 1.81	Reference		206	0.8 ± 1.22	Reference	
Iron	209	0.7 ± 1.70	7.2% (−3.7%, 19.4%)	0.20	206	0.8 ± 1.27	1.5% (−2.7%, 5.9%)	0.48
Cystatin C, mg/L								
Placebo	217	1.26 ± 0.29	Reference		205	2.06 ± 0.40	Reference	
Iron	216	1.25 ± 0.30	0.00 (−0.06, 0.05)	0.90	206	2.06 ± 0.37	0.00 (−0.07, 0.08)	0.97
eGFR,^2^ mL · min^−1^ · 1.73 m^−2^								
Placebo	217	62.1 ± 1.32	Reference		205	36.6 ± 1.18	Reference	
Iron	216	62.4 ± 1.32	0.5% (−4.6%, 5.8%)	0.86	206	36.6 ± 1.19	0.0% (−3.3%, 3.4%)	1.00
Hepcidin,^2^ µg/L								
Placebo	217	1.9 ± 3.41	Reference		207	8.1 ± 2.30	Reference	
Iron	216	4.4 ± 3.69	136.4% (86.1%, 200.3%)	<0.0005	207	9.1 ± 2.24	12.2% (−4.3%, 31.4%)	0.16
Hemoglobin, g/L								
Placebo	214	111.6 ± 19.0	Reference		209	150.6 ± 21.0	Reference	
Iron	215	120.7 ± 16.4	9.0 (5.7, 12.4)	<0.0005	206	153.8 ± 21.7	3.2 (−1.0, 7.3)	0.13
Anemia (hemoglobin < 110 g/L for mothers)								
Placebo	214	50.5 [108]	Reference				—	
Iron	215	21.4 [46]	−29.1% (−37.4%, −20.1%)	<0.001			—^3^	
Ferritin,^2^ µg/L								
Placebo	217	19.0 ± 2.61	Reference		205	103.0 ± 2.11	Reference	
Iron	216	37.1 ± 2.55	95.6% (63.6%, 133.9%)	<0.0005	206	127.0 ± 2.14	23.3% (6.6%, 42.7%)	0.005
Iron deficiency (ferritin ≤ 15 µg/L mothers and <12 µg/L for neonates)								
Placebo	217	43.3 [94]	Reference		205	0.9 [2]	Reference	
Iron	216	16.2 [35]	−27.1% (−35.0%, −18.7%)	<0.001	205	0.0 [0]	—^4^	ND
CRP, mg/L								
Placebo	217	6.7 ± 3.93	Reference		131	0.2 [0.2–0.3]	Reference	
Iron	216	7.7 ± 3.69	16.4% (−9.6%, 49.8%)	0.24	128	0.2 [0.2–0.3]	—^5^	0.62
Inflammation (CRP > 10 mg/L)								
Placebo	217	38.2 [83]	Reference		131	5.3 [7]	Reference	
Iron	216	39.8 [86]	1.6% (−7.6%, 10.7%)	0.74	128	1.6 [2]	−3.8% (−8.2%, 0.6%)	0.09

1 Values are mean ± SD or % [*n*] unless indicated otherwise. Effects are reported as absolute difference in means, relative difference (%) in geometric means, or difference in prevalence, with placebo as the reference group. Group estimates are medians [IQRs]; group differences in distributions were compared by independent-samples Mann–Whitney *U* test, which yields a *P* value only. For continuous outcomes, *P* values were obtained by simple linear regression analysis, accounting for heteroscedasticity. For binary outcomes, we used Newcombe's method to estimate 95% CIs and “N−1” chi-square tests to compute *P* values. We used log-binomial regression models to estimate prevalence differences when contingency tables contained cells with expected values <10. *P* values indicate the probability of data occurring as observed or being more extreme than observed under the assumption of no effect, i.e., outcomes being identically distributed for groups that received supplementation with either placebo or iron. β-Crosslaps, β-C-terminal telopeptide; CRP, C-reactive protein; eGFR, estimated glomerular filtration rate; intact-FGF23, intact fibroblast growth factor-23; ND, not determined; total-FGF23, intact and C-terminal fragments of fibroblast growth factor-23.

2 Geometric mean ± geometric SD.

3 Effects on anemia were not calculated in neonates because anemia is poorly defined in this group.

4 Not determined as there were too few cases of iron deficiency to allow analyses.

5 Plasma CRP concentration in cord blood was highly skewed and could not be normalized by log transformation.

Antenatal oral iron supplementation reduced the geometric mean neonatal total-FGF23 concentration (by 15.2%), whereas it increased the geometric mean neonatal intact-FGF23 concentration (by 21.6%) (Table 2). It improved neonatal iron stores (mean ferritin concentration increased by 23.3%) (Table 2) and other neonatal outcomes (birth weight, length at birth, and gestational duration increased by 141 g, 0.9 cm, and 3.4 d, respectively) described in our previous publication (12).

No marked group differences were seen in the other maternal or neonatal biomarkers of bone, kidney, or vitamin D metabolism. As might be expected with a randomized trial of this size, adjustment of treatment effects for baseline variables that were considered a priori to be prognostic for outcome did not markedly change effect estimates (**Supplemental Table 1**), showing that effects are attributable to the intervention alone and not to selection bias.

### Influence of baseline variables on intervention effects

**FIGURE 3:**
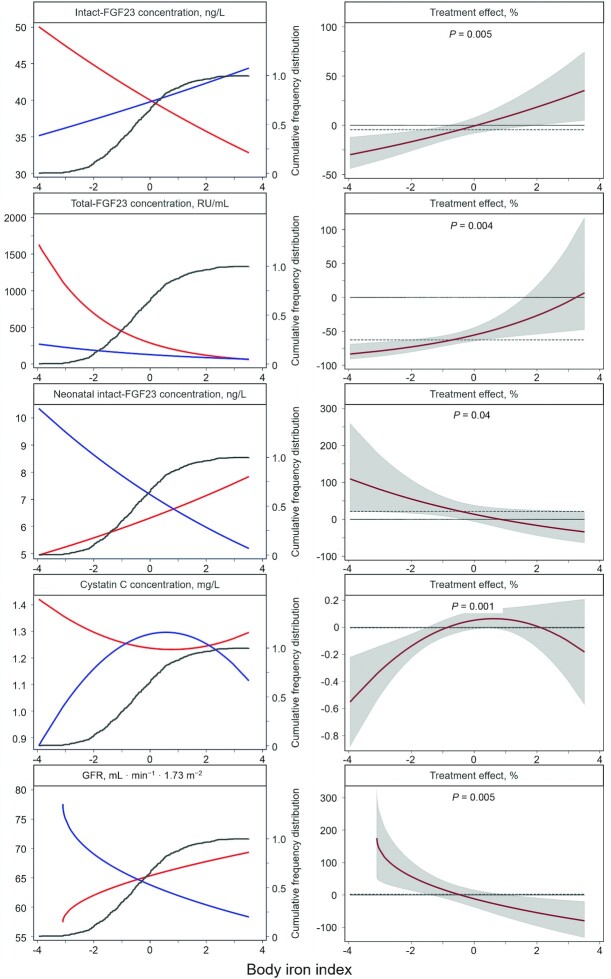
Effect of iron supplementation on selected outcomes at delivery, by iron status at baseline. Cumulative frequency distribution is represented by the gray line. The dependent variables are all maternal outcomes (iron, *n* = 216; placebo, *n* = 217) except for neonatal intact-FGF23 concentration (iron, *n* = 200; placebo, *n* = 205). Iron status is indicated by body iron index, i.e., the ln of the ratio of plasma concentrations of ferritin (µg/L) to plasma concentrations of soluble transferrin receptor (mg/L), both adjusted for plasma concentrations of C-reactive protein, α_1_-acid glycoprotein, and *Plasmodium* infection. *P* values were obtained by multiple fractional polynomial regression analysis, with adjustment for potentially influential maternal characteristics assessed at randomization, i.e., hemoglobin concentration, BMI, gestational age, parity, HIV infection, and *Plasmodium* infection. Left panels: associations between outcomes and body iron index for women who received supplementation with iron (blue lines) or placebo (red lines). The difference between these lines is the treatment effect (i.e., the relative difference in outcome between the iron group and the placebo group, with the placebo group used as the reference) conditional to body iron index. Right panels: treatment effect as a function of body iron index, with corresponding 95% confidence bands and *P* values. Horizontal dashed lines indicate zero effect and the unadjusted effect as measured in a regression model without covariates other than the intervention (Table 2). eGFR, estimated glomerular filtration rate; intact-FGF23, intact fibroblast growth factor-23; total-FGF23, intact and C-terminal fragments of fibroblast growth factor-23.

**Figure 3** shows intervention × body iron index interactions for selected outcomes. In women who received placebo (left panels, red lines), low iron status at baseline (as indicated by body iron index values) was associated with higher maternal concentrations of total-FGF23, intact-FGF23, and cystatin C, as well as lower neonatal intact-FGF23 concentration and lower maternal eGFR. Consistent with these observations, we found that antenatal oral iron supplementation reduced maternal total-FGF23 concentrations (overall: by 62.6%) (Table 2), with the magnitude of this effect being inversely associated with maternal iron status at baseline (Figure 3, second right panel; *P* = 0.004). Similarly, we found that antenatal oral iron supplementation increased neonatal intact-FGF23 concentration, with the magnitude of the effect being inversely associated with maternal iron status at baseline (*P* = 0.04).

For maternal intact-FGF23 concentration, maternal cystatin C concentration, and maternal eGFR, the effect of antenatal iron supplementation also varied by initial maternal iron status (Figure 3, right panels), but cumulative frequency distributions (Figure 3, left panels, gray lines) indicated that most women had body iron index values around the cutoffs that determined whether the effect of intervention was either positive or negative (intact-FGF23, eGFR) or body iron index values within the range of no effect (cystatin C). This may explain the absence of marked effects in the unadjusted analysis (Table 2; also indicated in Figure 3, right panels, dashed lines).

Analysis of effect modification by body iron index quantiles showed similar patterns as aforementioned (**Supplemental Figure 1**), indicating that the results presented in Figure 3 are not the result of overfitting. There was no evidence for effect modification for the other outcomes considered.

## Discussion

To our knowledge, we report the first randomized placebo-controlled trial to assess the effect of oral iron supplementation on FGF23 concentrations in persons without kidney disease. Antenatal iron supplementation reduced maternal total-FGF23 concentrations but not maternal intact-FGF23 concentrations at delivery. The magnitude of the effect on maternal total-FGF23 concentrations was inversely associated with initial maternal iron status. Iron supplementation decreased total-FGF23 concentration and increased intact-FGF23 concentration in neonates, and there was evidence that the magnitude and the direction of the intervention effect on neonatal intact-FGF23 concentration depended on initial maternal iron status. Although there was no measurable overall effect of iron supplementation on maternal intact-FGF23 concentration, maternal cystatin C concentration, and maternal eGFR, we found that initial maternal iron status may have influenced the direction and magnitude of these effects. Lastly, iron supplementation decreased maternal 25(OH)D concentration, but there was no evidence that this effect depended on initial maternal iron status (12).

The strengths of this study include its randomized design; large sample size; supervised administration of supplements with excellent adherence (iron: 100%; placebo: 99.1%) (12); blinding of participants, field staff, and laboratory staff to the intervention; the use of a placebo as a comparator; and the inclusion of iron-deficient and anemic women in the trial. One study limitation is that we assessed intervention effects on multiple endpoints, which has the inherent risk of multiplicity. On the other hand, effects on related outcomes can be mutually reinforcing instead of undermining (25). This point is underscored by our finding that iron supplementation led to increased maternal ferritin concentrations (Table 2), which lends credence to the concurrent increase in maternal hemoglobin concentrations: both findings indicate that the intervention improved maternal iron status. Results of the intervention × baseline body iron index interactions should be interpreted with caution and considered as exploratory because of the potential instability of the functions found, and because further studies are needed to validate the body iron index, adjusted for inflammation and infection, as an indicator of body iron status. In addition, in light of the decreases in 25(OH)D concentrations and the known effects of iron supplements on intestinal calcium absorption, it is likely that iron supplementation affects calcium metabolism. This finding could not be further investigated because blood samples were collected in EDTA-coated tubes which inhibits accurate calcium measurement. Plasma erythropoietin (EPO) concentration was not measured owing to constraints in available plasma volume and so could not be investigated in the causal pathway between iron supplementation and total-FGF23 reduction. An additional limitation is that certain characteristics that may have affected the outcome were not measured at baseline. However, with sample sizes >200/group, simple randomization normally yields only mild disparities in sample sizes between groups (26). Thus, it is unlikely that group imbalances in prognostic variables at baseline led to substantial selection bias in our effect estimates. The marked reduction in maternal and neonatal total-FGF23 concentrations after antenatal iron supplementation is in keeping with previous studies (6, 7) and is likely caused by a reversal of hypoxia and/or EPO-driven *FGF23* gene expression in osteocytes that occurs in iron deficiency (7). Consistent with this hypothesis, we found that the reduction in total-FGF23 was most pronounced in mothers with the lowest body iron status.

Our findings corroborate emerging evidence that orally administered iron does not normally lead to measurable changes in maternal plasma concentrations of intact-FGF23 or phosphate (6). Iron deficiency results in increased concentrations of hypoxia-inducible factor 1α, which stimulates *FGF23* transcription through increased EPO production and perhaps also by directly binding to the *FGF23* promoter (15). The resulting increase in FGF23 production does not normally result in maternal hypophosphatemia; excess FGF23 is proteolytically cleaved within osteocytes, resulting in increased secretion of C-terminal FGF23 fragments into the circulation (which is reflected in total-FGF23 concentrations), whereas concentrations of the biologically active intact hormone remain largely unaffected (27). By contrast, some intravenous iron preparations (e.g., ferric carboxymaltose, saccharated ferric oxide) appear to block cleavage of FGF23, thus leading to FGF23-mediated phosphaturia and a concomitant decrease in circulating phosphate concentrations (15). Although hypophosphatemia due to single-dose administration of intravenous iron is often transient and asymptomatic, it can persist for >1 mo, and some patients develop severe and symptomatic hypophosphatemia with serious musculoskeletal complications, including osteomalacia, fragility fractures, and hypoxemia (28).

In the present study, the effect of the iron intervention on maternal intact-FGF23 concentration varied with baseline iron status (Figure 3), suggesting that *FGF23* transcription and cleavage are uncoupled in women with poor iron status. In our study, this occurred in ∼40% of women (the lowest 2 quintiles; see Supplemental Figure 1). Chronically elevated concentrations of intact-FGF23 result in phosphaturia and eventually hypophosphatemia, which in turn can lead to serious musculoskeletal complications, including rickets in children and osteomalacia fragility fractures in adults. However, overall we found no evidence that iron affected plasma phosphate concentrations or bone turnover, although the intervention period (∼5 mo) may have been insufficient to detect changes in these markers. Further studies are needed to discount the possibility that moderate to severe iron deficiency causes hyperphosphaturia and hypophosphatemia in pregnant women. Special caution is warranted when prescribing intravenous ferric carboxymaltose to individuals with iron deficiency.

Our findings suggest that iron supplementation leads to increased glomerular filtration rates in women with poor iron status. This discovery is in agreement with a Mendelian randomization study that showed serum iron concentration to be associated with glomerular filtration rates (29). In a randomized controlled trial in Bangladesh, children whose mothers had antenatally received a daily oral supplement containing 60 mg Fe/d had higher glomerular filtration rates at 4.5 y of age than those whose mothers received 30 mg Fe/d (30). Children with a history of rickets had a reduced glomerular filtration rate as estimated from plasma cystatin C concentration (4). Further work is needed to elucidate a possible role of kidney function along the causal pathway between iron and rickets.

In a prospective cohort study, increased total-FGF23 concentration was associated with an increase in all-cause mortality in a general population of older US adults (31). Although this association may have been in part confounded by the effect of inflammation on FGF23 expression, it is possible that the reduction that is seen in maternal and infant total-FGF23 after antenatal iron supplementation confers a general health benefit.

Intriguingly, and in contrast to effects observed in pregnant women, antenatal iron supplementation led to *increased* intact-FGF23 concentrations in neonates, which conversely suggests that maternal iron deficiency during pregnancy leads to *decreased* neonatal intact-FGF23 concentrations, without an apparent effect on neonatal serum phosphate concentration. These findings contradict reports from a study in neonatal pups born to female wild-type mice which were fed an iron-deficient diet during the third week of pregnancy (corresponding to the human third trimester) and during lactation, and which developed increased concentrations of both intact-FGF23 and total-FGF23, as well as decreased phosphate concentrations (7). From that report (7), however, it is not clear whether these outcomes were measured immediately after birth.

In an earlier prospective cohort study, poor maternal iron status during pregnancy was associated with a higher total-FGF23 concentration but similar intact-FGF23 concentrations in infants and young children (6). Unfortunately, however, these markers were not assessed at delivery. In contrast to the critical role that FGF23 plays in regulating phosphorus and skeletal metabolism in adults and children, murine studies suggest that FGF23 may not be required to regulate fetal phosphorus homeostasis, placental phosphorus transport, or skeletal development, perhaps because the placenta's role to actively transport phosphorus and other minerals from the maternal circulation is independent of FGF23 (32). In mice, fetal phosphate concentrations are relatively high and decrease within a few days after birth, concurrently with the cessation of placental phosphate influx. Within hours to days after loss of the placental phosphorus pump, neonatal FGF23 becomes an important regulator of renal phosphorus excretion and intestinal phosphorus absorption (32). It is possible that the effects of antenatal iron supplementation on offspring FGF23 and phosphate metabolism may only become apparent later in infancy, and not at birth and so were not fully captured in this study.

Antenatal iron supplementation also had an effect on vitamin D metabolism by decreasing maternal 25(OH)D concentration. Emerging evidence has found a biological link between vitamin D and iron metabolism. The vitamin D response element has been identified on *HAMP* (the gene encoding hepcidin), and 1,25(OH)_2_D has been shown to suppress hepcidin expression and thereby increase the amount of iron absorbed from the diet and released from stores (33). It is possible that there is a negative feedback loop whereby when hepcidin concentrations are high (in iron repletion, or with antenatal iron supplementation or with inflammation), 25(OH)D concentration decreases. An alternative pathway for decreases in maternal 25(OH)D is through direct effects of FGF23 on vitamin D metabolism. Higher FGF23 concentrations are known to downregulate Cytochrome P450 Family 27 Subfamily B Member 1 (*CYP27B1*) expression and upregulate Cytochrome P450 Family 24 Subfamily A Member 1 (*CYP24A1*) expression with the potential to lower 1,25(OH)_2_D and raise 24,25(OH)_2_D, respectively (34).

We conclude that antenatal oral iron supplementation can redress perturbances in maternal and neonatal FGF23 metabolism induced by maternal iron deficiency during pregnancy. This suggests the possibility that iron supplementation could help prevent rickets and osteomalacia in individuals with an impaired ability to regulate FGF23 concentration.

## Supplementary Material

nqaa417_Supplemental_File

## Data Availability

Data described in the article may be made available upon request pending application and approval.

## Notes

Supported by Royal Society project grant RG2015R2 (to VSB) and the Medical Research Council (MRC) and the Department for International Development (DFID) under the MRC/DFID Concordat agreement via MRC programmes U105960371 (to AP) and MC-A760-5QX00 (to AMP). The original study was supported by the INSTAPA project which received funding from European Union Seventh Framework Programme FP7/2007/2013 grant 211484 (to MNM). KSJ is supported by National Institute for Health Research (NIHR) Cambridge Biomedical Research Centre grant IS-BRC-1215-20014. The NIHR Cambridge Biomedical Research Centre is a partnership between Cambridge University Hospitals NHS Foundation Trust and the University of Cambridge, funded by the NIHR. The views expressed are those of the authors and not necessarily those of the NHS, the NIHR, or the Department of Health and Social Care.

Supplemental Methods, Supplemental Table 1, and Supplemental Figure 1 are available from the “Supplementary data” link in the online posting of the article and from the same link in the online table of contents at https://academic.oup.com/ajcn/.

Abbreviations used: β-Crosslaps, β-C-terminal telopeptide; CRP, C-reactive protein; eGFR, estimated glomerular filtration rate; EPO, erythropoietin; FGF23, fibroblast growth factor-23; GSD, geometric SD; intact-FGF23, intact fibroblast growth factor-23 hormone; PTH, intact parathyroid hormone; sTfR, soluble transferrin receptor; total-FGF23, intact fibroblast growth factor-23 hormone and the C-terminal fragments that result from its cleavage; 1,25(OH)_2_D, 1,25-dihydroxyvitamin D; 25(OH)D, 25-hydroxyvitamin D.
