# Supplementary material for: Antenatal iron supplementation, FGF23, and bone metabolism in Kenyan women and their offspring: secondary analysis of a randomized controlled trial
Source: Am J Clin Nutr. 2021 Mar 1;113(5):1104–14. doi: 10.1093/ajcn/nqaa417 (PMC8106766; doi:10.1093/ajcn/nqaa417)
Supplement: nqaa417_Supplemental_File [file nqaa417_supplemental_file.docx]

**On-line Supplementary Material**

**Antenatal iron supplementation, FGF23 and bone metabolism in Kenyan women and their offspring: secondary analysis of a randomized controlled trial**

Vickie S. Braithwaite

**Supplementary methods: adjustment of plasma iron markers for inflammation**

Infections and inflammation can influence circulating marker concentrations (ferritin, soluble transferrin receptor) that are used to assess iron status. In a recent series of papers (Namaste et al. 2017a,b, Rohner et al. 2017, Mei et al. 2017, Larson et al. 2017), a group of researchers joined in the Biomarkers Reflecting Inflammation and Nutritional Determinants of Anemia (BRINDA) project proposed a method to adjust micronutrient status markers for inflammation (indicated by circulating concentrations of C-reactive protein, CRP, and *α*_1_-acid glycoprotein, AGP) and *Plasmodium* infection. In a recent review (unpublished), we identified several problems with this method. A key problem is that estimates of population micronutrient status are highly dependent on the choice of the reference values, especially when these values approach zero.

We compared log-transformation of inflammatory markers, as proposed by the BRINDA group, with alternative transformations (fractional polynomials, cube root transformation, inverse hyperbolic sine transformation) that have the advantages of being weaker at low values, and being defined at zero. We fit models using maximum likelihood methods and compared the balance between model fit and model simplicity using Akaike’s and Bayesian Information Criteria (AIC and BIC). Because CRP values were left-censored at the limit of quantification (1mg/L), we initially restricted the analysis to cases with non-censored values (415 of 470 women). For these cases, we adjusted ferritin concentration for *Plasmodium* infection and inflammatory markers (CRP and AGP, both cube root-transformed), using the following adjustment formula:

$$\text{}$$

$$\ln{Ferritin}_{adj}=\ln{Ferritin}_{unadj}-0.1859\cdot\sqrt[3]{{CRP}_{unadj}}-2.5031\cdot\sqrt[3]{{AGP}_{unadj}}- 0.4154 \cdot Plasmodium$$

where $Ferritin$, $CRP$ and $AGP$ indicate plasma concentrations of ferritin (μg/L), C-reactive protein (mg/L) and *α*_1_-acid glycoprotein (g/L) (all continuous variables), respectively; $Plasmodium$ is a binary indicator of the presence or absence of *Plasmodium* infection; and the suffixes *adj* and *unadj* denote adjusted and unadjusted values. Cube root transformation of inflammatory markers yielded a better model fit than with natural log transformation (AIC/BIC: 1,022/1,038 versus 1,018/1,035). We decided not to include reference values in our models because a) there is, to our knowledge, no evidence to support the assumption that micronutrient marker concentrations are independent of inflammatory markers in the range below the reference value (an assumption that is inherent to the BRINDA model); b) in bivariate analysis, ferritin models for AGP seemed to fit reasonably well in the AGP value range below the external reference value proposed by the BRINDA group (0.53g/L); c) the external reference value proposed by the BRINDA group for CRP (0.16 μg/L) is already very close to zero.

For 55 cases with left-censored CRP values, we fit a model and adjusted for AGP and *Plasmodium* infection only:

$$\ln{Ferritin}_{adj}=\ln{Ferritin}_{unadj}-1.1668\cdot\sqrt[3]{{AGP}_{unadj}}- 0.4048 \cdot Plasmodium$$

For soluble transferrin receptor concentrations, the best model included *Plasmodium* infection but not inflammatory markers:

$$\ln{sTfR}_{adj}=\ln{sTfR}_{unadj}- 0.1297 \cdot Plasmodium$$

where $sTfR$ indicates plasma concentrations of soluble transferrin receptor. There was no evidence that inflammatory markers were associated with soluble transferrin receptor concentrations independently of *Plasmodium* infection. This corroborates evidence from previous studies that circulating soluble transferrin receptor concentrations are independent of inflammation, but they are increased due to increased erythropoiesis induced by *Plasmodium* infection (Verhoef et al. 2001).

Lastly, we calculated the body iron index as follows:

$$Body iron index=\text{ln}\text{ }\left[ {{Ferritin concentration}_{adj}}/{{sTfR}_{adj}} \right]$$

In a small study, the *body iron index* (logarithm of the ratio of ferritin to sTfR) was shown to be linearly associated with quantitative estimates of the size of the body iron store in iron-replete individuals, or with the size of the functional deﬁcit that would need to be corrected before iron could again be accumulated in the store in iron-deﬁcient individuals (Skikne et al. 1992). The formula for this relation was validated in a nonrepresentative subset of healthy adult Caucasians participating in NHANES III (1988-1994), pregnant Jamaican women participating in an iron supplementation trial, and anemic Vietnamese women participating in an intervention trial with iron-fortiﬁed ﬁsh sauce (Pfeiffer et al. 2017).

**References**

Namaste SM, Rohner F, Huang J, Bhushan NL, Flores-Ayala R, Kupka R, Mei Z, Rawat R, Williams AM, Raiten DJ, Northrop-Clewes CA, Suchdev PS. Adjusting ferritin concentrations for inflammation: Biomarkers Reflecting Inflammation and Nutritional Determinants of Anemia (BRINDA) project. *Am J Clin Nutr* 2017; **106(Suppl 1):** 359S–71S

Pfeiffer CM, Looker AC. Laboratory methodologies for indicators of iron status: strengths, limitations, and analytical challenges. *Am J Clin Nutr* 2017; **106(Suppl 6):** 1606S–14S.

Rohner F, Namaste SM, Larson LM, Addo OY, Mei Z, Suchdev PS, Williams AM, Sakr Ashour FA, Rawat R, Raiten DJ, Northrop-Clewes CA. Adjusting soluble transferrin receptor concentrations for inflammation: Biomarkers Reflecting Inflammation and Nutritional Determinants of Anemia (BRINDA) project. *Am J Clin Nutr* 2017; **106(Suppl 1):** 372S–82S.

Skikne BS, Flowers CH, Cook JD. Serum transferrin receptor: a quantitative measure of tissue iron deficiency. *Blood* 1990; **75:** 1870–76.

Verhoef H, West CE, Ndeto P, Burema J, Beguin Y, Kok FJ. Serum transferrin receptor concentration indicates increased erythropoiesis in Kenyan children with asymptomatic malaria. *Am J Clin Nutr* 2001; **74:** 767–75.

**Supplementary table 1. Intervention effects on selected outcomes at delivery, with and without adjustment * for baseline variables**

| **Outcome** | n | **Group descriptives †** | **Effect (95% CI), unadjusted** | **P-value** | **Effect (95% CI), adjusted** | **P-value** |
| --- | --- | --- | --- | --- | --- | --- |
| **Maternal blood concentration** |  |  |  |  |  |  |
| **Total-FGF23, RU/mL** ‡ |  |  |  |  |  |  |
| Placebo | 217 | 370.3 [3.62] | Reference |  | Reference |  |
| Iron | 216 | 138.4 [3.09] | -62.6% (-70.3%, -53.0%) | <0.001 | -60.2% (-68.1%, -50.5%) | <0.0005 |
| **Intact-FGF23, ng/L** ‡ |  |  |  |  |  |  |
| Placebo | 217 | 41.3 [1.54] | Reference |  | Reference |  |
| Iron | 216 | 39.4 [1.46] | -4.5% (-11.5%, 3.1%) | 0.24 | -5.5% (-12.6%, 2.2%) | 0.16 |
| **25-hydroxyvitamin D, nmol/L** |  |  |  |  |  |  |
| Placebo | 217 | 99.6 (28.7) | Reference |  | Reference |  |
| Iron | 216 | 93.5 (23.0) | -6.1 (-11.0, -1.2) | 0.02 | -6.9 (-11.9, -1.8) | 0.008 |
| **Vitamin D insufficiency (25-hydroxyvitamin D < 50 nmol/L)** | | | | |  |  |
| **Placebo** | 217 | 2.8% [6] | Reference |  | Reference |  |
| **Iron** | 216 | 3.7% [8] | 0.9% (-2.4% to 4.3%) | 0..58 | 1.0% (-2.4% to 4.4%) | 0.56 |
| **1,25-dihydroxyvitamin D, pmol/L** |  |  |  |  |  |  |
| Placebo | 203 | 351.0 (78.2) | Reference |  | Reference |  |
| Iron | 200 | 351.0 (84.4) | 0.1 (-15.8, 16.1) | 0.99 | 3.5 (-12.9, 19.8) | 0.67 |
| **Parathyroid hormone, pmol/L** ‡ |  |  |  |  |  |  |
| Placebo | 217 | 4.0 [1.94] | Reference |  | Reference |  |
| Iron | 216 | 3.9 [1.92] | -2.0% (-13.4%, 10.9%) | 0.75 | -1.9% (-13.7%, 11.5%) | 0.77 |
| **Phosphate, mmol/L** |  |  |  |  |  |  |
| Placebo | 217 | 1.27 (0.32) | Reference |  | Reference |  |
| Iron | 216 | 1.29 (0.24) | 0.03 (-0.03, 0.08) | 0.30 | 0.01 (-0.04, 0.07) | 0.63 |
| **Total alkaline phosphatase, U/L** ‡ |  |  |  |  |  |  |
| Placebo | 217 | 102.3 [1.88] | Reference |  | Reference |  |
| Iron | 215 | 96.1 [1.86] | -6.0% (-16.5%, 5.7%) | 0.30 | -2.9% (-13.8, 9.4%) | 0.63 |
| **β-Crosslaps**, **µg/L** ‡ |  |  |  |  |  |  |
| Placebo | 210 | 0.61 [1.81] | Reference |  | Reference |  |
| Iron | 209 | 0.66 [1.70] | 7.2% (-3.7%, 19.4%) | 0.20 | 8.6% (-2.4%, 20.8%) | 0.13 |
| **Cystatin C, mg/L** |  |  |  |  |  |  |
| Placebo | 217 | 1.26 (0.29) | Reference |  | Reference |  |
| Iron | 216 | 1.25 (0.30) | 0.00 (-0.06, 0.05) | 0.90 | -0.01 (-0.07, 0.04) | 0.66 |
| **eGFR, mL/min/1.73m^2^** |  |  |  |  |  |  |
| Placebo | 217 | 62.1 (17.4) | Reference |  | Reference |  |
| Iron | 216 | 64.4 (17.0) | 0.5% (-4.6% to 5.8%) | 0.86 | 0.8% (-4.3%, 6.3%) | 0.76 |
| **Hepcidin, µg/L** ‡ |  |  |  |  |  |  |
| Placebo | 217 | 1.9 [3.41] | Reference |  | Reference |  |
| Iron | 216 | 4.4 [3.69] | 136.4% (86.1%, 200.3%) | <0.001 | 126.0% (76.2%, 189.8%) | <0.0005 |
| **Hemoglobin, g/L** |  |  |  |  |  |  |
| Placebo | 214 | 111.6 (19.0) | Reference |  | Reference |  |
| Iron | 215 | 120.7 (16.4) | 9.1 (5.7, 12.4) | <0.001 | 7.8 (4.6, 11.1) | <0.0005 |
| **Anemia (hemoglobin <110 g/L)** |  |  |  |  |  |  |
| Placebo | 214 | 50.4% [108] | Reference |  | Reference |  |
| Iron | 215 | 21.4% [46] | -29.1% (-37.4%, -20.1%) | <0.001 | -26.6% (-34.7%, -18.5%) | <0.0005 |
| **Ferritin, µg/L** ‡ |  |  |  |  |  |  |
| Placebo | 217 | 19.0 [2.61] | Reference |  | Reference |  |
| Iron | 216 | 37.1 [2.55] | 95.6% (63.6%, 133.9%) | <0.001 | 91.3% (60.8%, 127.5%) | <0.0005 |
| **Iron deficiency (Ferritin** **≤15µg/L**) |  |  |  |  |  |  |
| Placebo | 217 | 43.3% [94] | Reference |  | Reference |  |
| Iron | 216 | 16.2% [35] | -27.1% (-35.0%, -18.7%) | <0.001 | -25.7% (-33.8%, -17.6%) | <0.0005 |
| **C-reactive protein, mg/L** ‡ |  |  |  |  |  |  |
| Placebo | 217 | 6.7 [3.93] | Reference |  | Reference |  |
| Iron | 216 | 7.8 [3.69] | 16.4% (-9.6%, 49.8%) | 0.24 | 24.0% (-3.5%, 59.2%) | 0.09 |
| **Inflammation (C-reactive protein >10 mg/L)** |  |  |  |  |  |  |
| Placebo | 217 | 38.2% [83] | Reference |  | Reference |  |
| Iron | 216 | 39.8% [86] | 1.6% (-7.6%, 10.7%) | 0.74 | 3.4% (-5.7%, 12.4%) | 0.47 |
| **Neonatal (cord) blood concentration** |  |  |  |  |  |  |
| **Total-FGF23, RU/mL** ‡ |  |  |  |  |  |  |
| Placebo | 207 | 647.3 [2.29] | Reference |  | Reference |  |
| Iron | 207 | 548.6 [2.50] | -15.2% (-28.4%, 0.3%) | 0.06 | -9.5% (-23.8%, 7.4%) | 0.25 |
| **Intact-FGF23, ng/L** ‡ |  |  |  |  |  |  |
| Placebo | 205 | 6.1 [2.65] | Reference |  | Reference |  |
| Iron | 200 | 7.4 [2.47] | 21.6% (1.2%, 46.1%) | 0.04 | 22.4% (1.3%, 47.8%) | 0.04 |
| **25-hydroxyvitamin D, nmol/L** |  |  |  |  |  |  |
| Placebo | 204 | 63.1 (19.3) | Reference |  | Reference |  |
| Iron | 206 | 61.5 (17.7) | -1.6 (-5.2, 2.0) | 0.37 | -2.2 (-6.0, 1.5) | 0.24 |
| **Vitamin D insufficiency (25-hydroxyvitamin D < 50 nmol/L)** | | | | |  |  |
| **Placebo** | 204 | 23.5% [48] | Reference |  | Reference |  |
| **Iron** | 206 | 25.2% [52] | 1.7% (-6.9, 10.3) | 0.69 | 2.1 (-5.4, 9.48) | 0.59 |
| **1,25-dihydroxyvitamin D, pmol/L** |  |  |  |  |  |  |
| Placebo | 195 | 203.0 (62.9) | Reference |  | Reference |  |
| Iron | 195 | 203.9 (52.7) | 0.9 (-10.7, 12.4) | 0.88 | 2.3 (-9.6, 14.2) | 0.71 |
| **Parathyroid hormone, pmol/L** ‡ |  |  |  |  |  |  |
| Placebo | 205 | 0.5 [2.69] | Reference |  | Reference |  |
| Iron | 206 | 0.5 [2.97] | -3.3% (-21.0%, 18.3%) | 0.74 | -0.8% (-19.3%, 22.1%) | 0.94 |
| **Phosphate, mmol/L** |  |  |  |  |  |  |
| Placebo | 205 | 2.05 (0.64) | Reference |  | Reference |  |
| Iron | 206 | 2.11 (0.65) | 0.06 (-0.07, 0.18) | 0.38 | 0.05 (-0.08, 0.17) | 0.46 |
| **Total alkaline phosphatase, U/L** ‡ |  |  |  |  |  |  |
| Placebo | 205 | 17.3 [1.74] | Reference |  | Reference |  |
| Iron | 206 | 17.6 [2.22] | 2.0% (-10.9%, 16.8%) | 0.77 | 2.1% (-10.9%, 17.1%) | 0.76 |
| **β-Crosslaps**, **µg/L** ‡ |  |  |  |  |  |  |
| Placebo | 206 | 0.8 [1.22] | Reference |  | Reference |  |
| Iron | 206 | 0.8 [1.27] | 1.5% (-2.7%, 5.9%) | 0.48 | 1.6% (-2.7%, 6.1%) | 0.47 |
| **Cystatin C, mg/L** |  |  |  |  |  |  |
| Placebo | 205 | 2.06 (0.40) | Reference |  | Reference |  |
| Iron | 206 | 2.06 (0.37) | 0.00 (-0.07, 0.08) | 0.98 | 0.00 (-0.07, 0.08) | 0.92 |
| **eGFR,** **mL/min/1.73m^2^** ‡ |  |  |  |  |  |  |
| Placebo | 205 | 36.6 [1.18] | Reference |  | Reference |  |
| Iron | 206 | 36.6 [1.19] | 0.0% (-3.3%, 3.4%) | 1.00 | -0.1% (-3.5%, 3.4%) | 0.95 |
| **Hepcidin, µg/L** ‡ |  |  |  |  |  |  |
| Placebo | 207 | 8.1 [2.30] | Reference |  | Reference |  |
| Iron | 207 | 9.1 [2.24] | 12.2% (-4.3%, 31.4%) | 0.16 | 11.4% (-5.0%, 30.7%) | 0.18 |
| **Hemoglobin, g/L** |  |  |  |  |  |  |
| Placebo | 209 | 150.6 (21.0) | Reference |  | Reference |  |
| Iron | 206 | 153.8 (21.7) | 3.2 (-1.0, 7.3) | 0.13 | 3.9 (-0.2, 8.1) | 0.06 |
| **Ferritin, µg/L** ‡ |  |  |  |  |  |  |
| Placebo | 205 | 103.0 [2.11] | Reference |  | Reference |  |
| Iron | 206 | 127.0 [2.14] | 23.3% (6.6%, 42.7%) | 0.005 | 22.2% (5.1%, 42.1%) | 0.009 |
| **Iron deficiency (Ferritin <12 µg/L)** |  |  |  |  |  |  |
| Placebo | 205 | 0.9% [2] | Reference |  | Reference |  |
| Iron | 205 | 0.0% [0] | ND ‖ | ND | ND ‖ | ND |
| **C-reactive protein, mg/L** ‡ |  |  |  |  |  |  |
| Placebo | 131 | 0.2 [0.2, 0.2] **¶** | Reference |  | Reference |  |
| Iron | 128 | 0.2 [0.2, 0.3] **¶** | ND ****** | 0.62 | ND ****** | ND ‖ |
| **Inflammation (C-reactive protein >10 mg/L)** |  |  |  |  |  |  |
| Placebo | 131 | 5.3% [7] | Reference |  | Reference |  |
| Iron | 128 | 1.6% [2] | -3.8% (8.2%, 0.6%) | 0.09 | 8.5% (0.5%, 16.4%) | 0.04 |
| **Other neonatal outcomes** |  |  |  |  |  |  |
| **Length, cm** |  |  |  |  |  |  |
| Placebo | 197 | 49.6 (4.3) | Reference |  | Reference |  |
| Iron | 197 | 50.6 (4.2) | 0.9 (0.1, 1.8) | 0.03 | 0.8 (-0.1, 1.6) | 0.09 |
| **Length for gestational age and sex z-score, SD** ‡ |  |  |  |  |  |  |
| Placebo | 194 | 0.57 [2.36] | Reference |  | Reference |  |
| Iron | 197 ****** | 0.91 [2.46] | 0.34 (-0.14, 0.82) | 0.16 | 0.19 (-0.29, 0.69) | 0.43 |
| **Weight, g** |  |  |  |  |  |  |
| Placebo | 199 | 3,050 (410) | Reference |  | Reference |  |
| Iron | 194 | 3,191 (441) | 140.7 (56.3, 225.1) | 0.001 | 121.3 (37.2, 205.4) | 0.005 |
| **Weight for gestational age z-score, SD** |  |  |  |  |  |  |
| Placebo | 197 | -0.14 (0.91) | Reference |  | Reference |  |
| Iron | 194 | 0.02 (1.04) | 0.17 (-0.03, 0.36) | 0.09 | 0.10 (-0.09, 0.29) | 0.30 |
| **Head circumference, cm** ‡ |  |  |  |  |  |  |
| Placebo | 197 ‡‡ | 34.6 (1.06) | Reference |  | Reference |  |
| Iron | 197 | 34.9 (1.05) | 0.9% (-0.1%, 1.9%) | 0.08 | 0.7% (-0.3%, 1.8%) | 0.19 |
| **Head circumference z-score, SD** |  |  |  |  |  |  |
| Placebo | 194 | 0.97 (1.65) | Reference |  | Reference |  |
| Iron | 197 | 1.06 (1.40) | 0.10 (-0.21, 0.40) | 0.53 | 0.01 (-0.30, 0.33) | 0.94 |
| **Gestational age, days** |  |  |  |  |  |  |
| Placebo | 229 | 271.0 (15.2) | Reference |  | Reference |  |
| Iron | 235 | 274.4 (12.7) | 3.4 (0.9, 6.0) | 0.01 | 3.5 (1.0, 6.1) | 0.007 |

eGFR: estimated glomerular filtration rate; FGF: fibroblast growth factor; ND: not determined.

* Adjusted for maternal hemoglobin concentration, body iron index, age, body mass index, gestational age (ultrasound), parity, infection status for HIV and *Plasmodium* infection (all species, by rapid dipstick test and quantitative PCR assay), all assessed at randomisation. † Values are mean (SD), ‡ geometric mean (GSD) or prevalence [n]. Effects are reported as absolute difference in means, relative difference (%) in geometric means, with placebo as the reference group, or difference in prevalence. Not determined because ‖ there were too few cases of iron deficiency to allow analyzes, and because ** plasma C-reactive protein concentration in cord blood was highly skewed and could not be normalised by log-transformation. ¶ Group estimates are medians [25^th^ and 75^th^ percentiles]; group differences were compared by independent samples Mann-Whitney U test, which yields a p-value only. Excluding single outliers at ** -29.35 SD and ‡‡ 0.

**Supplementary Figure 1.**

**Effect of iron supplementation on selected outcomes at delivery, by body iron index quintile**

Body iron index: the natural logarithm of the ratio of plasma concentrations of ferritin (µg/L) and soluble transferrin receptor (mg/L), both adjusted for plasma concentrations of C-reactive protein, *α*_1_-acid glycoprotein and *Plasmodium* infection (see text); Total-FGF23: C-terminal fragments and intact fibroblast growth factor-23; eGFR: estimated glomerular filtration rate; Intact-FGF23: intact fibroblast growth factor-23; Q1-Q5: quintile 1: <0.24; quantile 2: 0.24-0.84; quantile 3: 0.84-1.42; quantile 4: 1.42-2.01; quantile 5: ≥2.01.

Maternal outcomes unless indicated otherwise. Effect estimates were estimated by multiple linear regression models with main effect terms for intervention and dummy-coded quintiles for body iron index, and their product terms, without adjustment for baseline variables. Regression coefficients thus obtained were used to calculate marginal effects. Body iron index was computed as the natural logarithm of the ratio of plasma concentrations of ferritin (µg/L) and soluble transferrin receptor (mg/L), both adjusted for plasma concentrations of C-reactive protein, *α*_1_-acid glycoprotein and *Plasmodium* infection (see main text). Line bars indicate 95% CIs.
